# Molecular Phylogenetics and Morphological Analyses Support *Dolichopoda*, a New Neotropical Genus of Marantaceae (Zingiberales)

**DOI:** 10.3390/plants14223486

**Published:** 2025-11-15

**Authors:** Naédja K. M. Luna, Thales S. Coutinho, Mark W. Chase, Leonardo P. Felix, Edlley M. Pessoa

**Affiliations:** 1 Programa de Pós-graduação em Biologia Vegetal, Departamento de Botânica, Universidade Federal de Pernambuco, Recife 50670-901, Pernambuco, Brazil; 2 Departamento de Botânica e Ecologia, Instituto de Biociências, Universidade Federal de Mato Grosso, Cuiabá 78060-900, Mato Grosso, Brazil; thales_scoutinho@hotmail.com; 3 Royal Botanic Gardens Kew, Richmond TW9 3AE, UK; 4 Centro de Ciências Agrárias, Departamento de Biociências, Universidade Federal da Paraíba, Areia 58051-900, Paraíba, Brazil; 5 Centro de Ciências Naturais e Humanas, Universidade Federal do ABC, Santo André 09280-560, São Paulo, Brazil; edlley.pessoa@ufabc.edu.br

**Keywords:** Ancestral state reconstruction, *Ctenanthe*, *Maranta* clade, *Saranthe*, *Stromanthe*

## Abstract

This study presents a molecular phylogenetic analysis based on four DNA regions (plastid *matK*, *trnL-trnF*, and *rps16* and nuclear ribosomal ITS) for *Ctenanthe*, *Saranthe* and *Stromanthe*, encompassing nearly 70% of species in these genera. Given this extensive sampling, we assess the monophyly of these genera and evaluate whether key morphological traits can serve as diagnostic characters (synapomorphies). For the first time, we included *Stromanthe bahiensis*, an unusual species that differs from all congeneric species in its elongate petioles and relatively long, pendulous, hirsute synflorescences. Our findings reveal *S. bahiensis* as sister to the rest of the group. The evolution of key morphological traits (organization of the aerial shoot system, rachis flexuosity, spathe compactness and cymule type) were estimated to be complex, with none exhibiting consistent diagnostic utility. Given that these traits were among the main reasons *S. bahiensis* was described under *Stromanthe*, our ancestral state reconstruction, coupled with its phylogenetic position, justifies recognition of a new genus, *Dolichopoda*. Our findings suggest that morphological evolution in the group may have been shaped by convergence, parallelisms and reversals, which may partially explain the high morphological overlap observed among genera. This realignment not only resolves phylogenetic inconsistencies but also facilitates more accurate biogeographic and ecological inferences. Additionally, we transfer *Ctenanthe dasycarpa* to *Stromanthe* to make *Ctenanthe* and *Stromanthe* mutually monophyletic.

## 1. Introduction

Marantaceae are the second largest family in the Zingiberales, with 28 genera and 588 species [1,2,3,4,5]. They have a Pantropical distribution but are more diverse in the Neotropics, where 15 genera and about 450 species have been recorded. Their richness is especially high in the Amazon Rainforest and the Atlantic Forest, where they are important components of the understory [1,5,6]. Compared to the other Zingiberales, the family is exceptionally variable in floral and vegetative morphology, representing a good example of adaptative radiation in the Neotropics [1,3,6,7,8]. Andersson [1] proposed a classification of five informal generic groups based on reproductive features: the *Prynium*, *Calathea*, *Donax*, *Maranta* and *Myrosma* groups. Then, combining morphological and molecular data (plastid *rps16*), Andersson and Chase [7] confirmed these groups, whereas Prince and Kress [8], using only molecular data (plastid *trnL-F* and *matK*) and a better species sampling, identified five clades, of which two were not the same as in Andersson and Chase [7]: the *Sarcophrynium* and *Stachyphrynium* clades (replacing the *Prynium* and *Myrosma* clades *sensu* Andersson and Chase). Subsequent phylogenetic studies using nuclear and plastid markers [9,10] confirmed the results of Prince and Kress [8]. Transition from morphology-based to molecular phylogenetic classification refined, but at this point did not fully stabilize, the generic limits in Marantaceae due to sampling and limitations of the molecular markers used.

The *Maranta* clade is the second most diverse in the family after that of *Calathea* [5], but this group remains one of the most in need of further study due to its high diversity, broad geographic distribution, and poorly resolved phylogenetic relationships. It is strongly supported and currently includes the Neotropical genera *Ctenanthe* Eichler (16 species), *Maranta* Plum. ex L. s.l. (51 species), *Saranthe* (Regel & Körn.) Eicher (10 species), *Stromanthe* Sond. (20 species), and Paleotropical *Halopegia* K.Schum. (3 species) and *Indianthus* Suksathan & Borchs. (1 species) [4,5,8,9].

**Figure 1:**
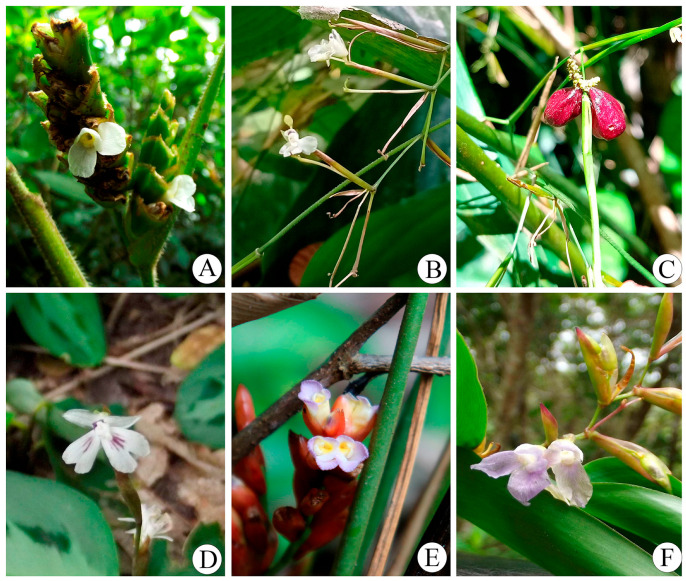
Neotropical genera of the *Maranta* clade. (**A**) *Ctenanthe*. (**B**) *Dolichopoda*: synflorescence. (**C**) *Dolichopoda*: fruit. (**D**) *Maranta*. (**E**) *Saranthe*. (**F**) *Stromanthe*.

The main morphological features characterizing the *Maranta* clade are absent bracteoles, two outer staminodes (rarely one in *Stromanthe*), one callose staminode, one cucullate staminode and dehiscent capsules (indehiscent only in *Halopegia*) [8]. Fernandes et al. [3] analyzed the *Maranta* clade, in which *Maranta* was enlarged to include *Hylaeanthe* A.M.E.Jonker & Jonker, *Koernickanthe* L.Andersson and *Myrosma* L.f. and confirmed *Maranta* s.l. as sister to *Ctenanthe*, *Saranthe* and *Stromanthe* (Figure 1). Fernandes et al. [3] found *Ctenanthe* and *Stromanthe* to be non-monophyletic, corroborating previous studies [8,9,10]. However, sampling of these genera as well as *Saranthe* was low (around 25% of the species in the three genera), which limits the resolution of infrageneric relationships and prevents establishment of more robust generic delimitation. Thus, there was insufficient taxon coverage to evaluate the monophyly of these genera or evolutionary significance of key morphological traits.

Because several genera in the *Maranta* clade have been consistently recovered as non-monophyletic in previous studies, we provide a new analysis focused on *Ctenanthe*, *Saranthe* and *Stromanthe*, in which we expand the sampling to nearly 70% of the species in these genera. Importantly, we include for the first time the morphologically aberrant, microendemic *Stromanthe bahiensis* Yoshida-Arns, Mayo & J.M.A.Braga, which is uniquely characterized by much longer petioles and much longer pendulous hirsute synflorescences [11]. Including it in the sampling is crucial for assessing the infrageneric boundaries in this clade, as it possesses a unique morphology that contributes to a more precise understanding of diversity and evolution in this group. Here, we follow an integrative approach assessing monophyly of these genera and trait evolution to evaluate whether key morphological traits can serve as diagnostic characters (synapomorphies) or reflect homoplasy or plesiomorphy. Based on these results, we propose revised circumscriptions for these taxa.

## 2. Results

### 2.1. Phylogenetic Relationships

We generated 68 new sequences for Marantaceae. Our complete matrix was 4133 bp long: nrITS matrix 681 bp with 17.5% being potentially parsimony-informative; *matK* 1602 bp with 2.3% potentially parsimony-informative; *rps16* 902 bp with 5.4% potentially parsimony-informative; and *trnL-F* 948 bp, with 1.5% potentially parsimony-informative sites (Table 1). All accessions included in the analyses are listed in Table S1. Although plastid regions showed relatively low variability, the nuclear ribosomal (nr)ITS region provided substantial phylogenetic signal, supporting the combined approach.

The nrITS tree recovered *Maranta* s.l as four unsupported successively diverging clades (PP < 0.7). However, *Ctenanthe*, *Saranthe* and *Stromanthe* were recovered in a strongly supported clade (posterior probability, PP 1.00, Figure S1A). In this last clade, *Stromanthe bahiensis* was sister to the other species of these three genera, albeit without support (PP < 0.7). The other species of *Stromanthe* were in a strongly supported clade (Figure S1A, PP 1.00). Most species of *Ctenanthe*, except *C. muelleri* Petersen, were also in a strongly supported clade (Figure S1A, PP: 1.00). Only *Saranthe* was supported as monophyletic (Figure S1A, PP 1.00).

The combined plastid tree recovered *Maranta* s.l. as two successively diverging clades, both strongly supported (Figure S1B, PP 1.00), but with their inter-relationships unsupported. *Ctenanthe*, *Saranthe* and *Stromanthe* were recovered in similar positions as in the nrITS tree, except *Saranthe klotzchiana* (Koer.) Eichl., which was in a polytomy rather than in a clade with its congeners. *Ctenanthe dasycarpa* (Donn.Sm.) K.Schum., not sampled for nrITS, was included in *Stromanthe* with strong support (Figure S1B, PP 0.99).

**Figure 2:**
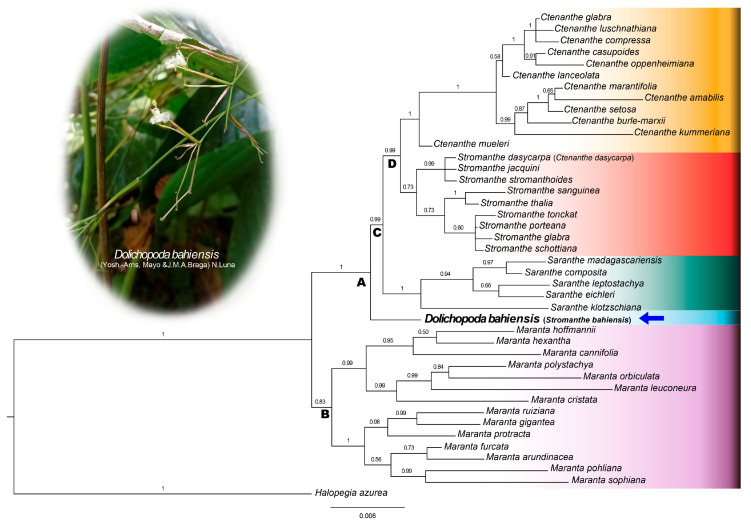
Phylogenetic relationships of Neotropical genera of the *Maranta* clade from Bayesian inference of one nuclear (rITS) and three plastid regions (*rps16* intron, *trnL-F* intron and spacer and *matK* exon). Posterior probabilities are shown above the branches.

Since no strongly supported inconsistencies were found in the nrITS and combined plastid trees (Figure S1), we performed a combined analysis (Figure 2). Clade A includes *Ctenanthe*, *Saranthe* and *Stromanthe* (Figure 2, PP 1.00). This clade is sister to clade B (PP < 0.7), which included *Maranta* sl. (Figure 2, PP 0.83). In clade A, *Stromanthe bahiensis* was supported as sister to clade C, which includes all other species (Figure 2, PP 0.99). Clade C included the species of *Saranthe* (PP 1.00) as sister to clade D (PP 0.99), comprising *Ctenanthe* and *Stromanthe*. Both *Ctenanthe* and *Stromanthe* were found to be non-monophyletic. Most species of *Stromanthe* are in a separate clade (except to *S. bahiensis*, Figure 2, PP 0.73), whereas most of *Ctenanthe* are in the other clade (Figure 2, PP 1.00), except for *C. dasycarpa*, which is a member of a strongly supported clade with *S. jacquini* and *S. stromanthoides* (Figure 2, PP 0.99).

In all trees, the clade formed by *Ctenanthe*, *Saranthe* and *Stromanthe* was strongly supported. *Stromanthe bahiensis* was recovered as an isolated species in all trees, supporting the need for taxonomic changes to accommodate it. All trees strongly supported non-monophyly of *Ctenanthe* and *Stromanthe*, reinforcing the need for their taxonomic recircumscription.

### 2.2. Ancestral State Evolution

**Figure 3:**
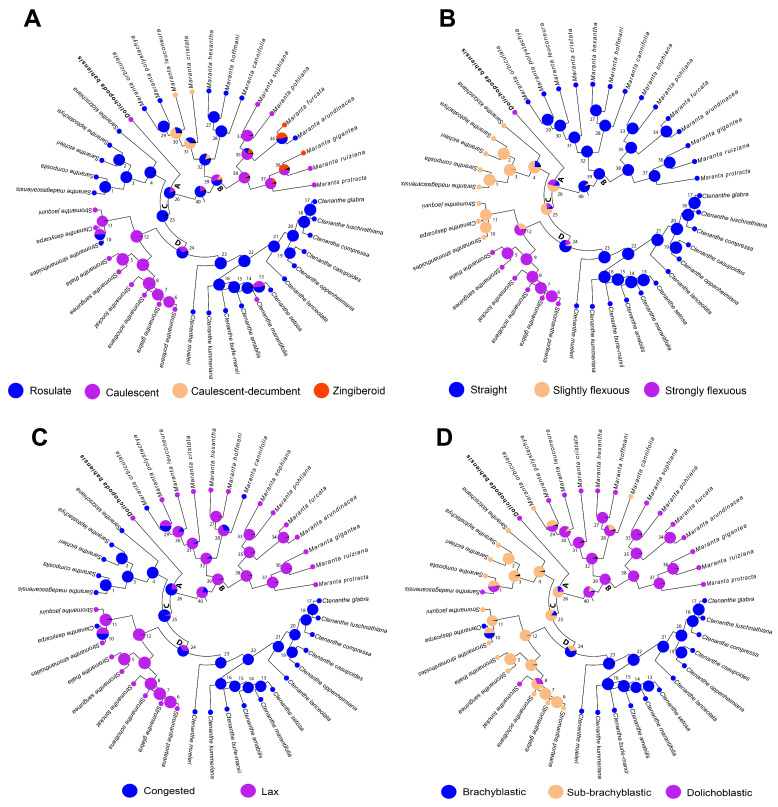
Ancestral state reconstructions for Neotropical members of the *Maranta* clade produced using APE for (**A**) organization of the aerial shoot system; (**B**) rachis shape; (**C**) spathe compactness; (**D**) cymule type.

In our analyses, the rosulate aerial shoot system was reconstructed as plesiomorphic (node 40, 73.77%; Figure 3A, Table S4), whereas the caulescent aerial shoot system exhibited homoplasy, arising independently at least four times (node 12 = 98.53% and 38 = 89.04%, as well as in *Ctenanthe marantifolia* (Vell.) J.M.A.Braga & H.Gomes and *Stromanthe bahiensis*; Figure 3A, Table S4). The ancestral rachis flexuosity for the entire group was inferred as straight (node 40 = 88.09%; Figure 3B, Table S4). In contrast, the most recent common ancestor of the clade comprising *Ctenanthe*, *Saranthe* and *Stromanthe* was reconstructed as having a slightly flexuous rachis (node 26 = 54.29%; Figure 3B, Table S4). The strongly flexuous rachis was also homoplasious, appearing independently at least twice (node 12 = 66.23% and in *Stromanthe bahiensis*; Figure 3B, Table S4). A florescence with lax spathes was reconstructed as plesiomorphic (node 40 = 80.75%; Figure 3C, Table S4), whereas the congested form arose multiple times independently. The dolichoblastic cymule was inferred as the ancestral state (node 40 = 76.79%; Figure 3D, Table S4), whereas the most recent common ancestor of *Ctenanthe*, *Saranthe* and *Stromanthe* likely had a sub-brachyblastic cymule (node 26 = 73.40%; Figure 3D, Table S4). At least three reversions to the dolichoblastic state were observed in *Saranthe madagascariensis*, *Stromanthe tonckat* (Aubl.) Eichler and *Stromanthe bahiensis*. Although the ancestral state reconstructions yielded high probabilities, we recognize that these estimates can be sensitive to taxon sampling (eg. for *Stromanthe*) and character coding. In summary, the four traits that led to the original placement of *S. bahiensis* under *Stromanthe* are plesiomorphic or homoplastic.

## 3. Discussion

Our results confirmed the clade formed by *Ctenanthe*, *Saranthe* and *Stromanthe* as sister to *Maranta*, in agreement with previous studies [3,10]. Reconstruction of ancestral morphological states revealed that four characters traditionally used to diagnose genera of the *Maranta* clade are plesiomorphic or homoplastic (Figure 3), providing a clearer understanding of why previous morphological classifications [12] failed to reflect evolutionary relationships. The rosulate aerial shoot system, inferred as ancestral for the group, was retained in multiple clades, whereas the caulescent habit, as in *S. bahiensis*, appeared independently at least four times. Likewise, rachis flexuosity, cymule type and spathe compactness exhibited independent transitions. The patterns observed suggest that morphological shifts are evolutionarily labile in the *Maranta* clade, possibly linked to ecological transitions [13], reinforcing their unreliability for generic delimitation. Importantly, *S. bahiensis* combines several of these ancestral or homoplastic states, explaining its spurious placement under *Stromanthe*. Future analyses including additional anatomical and ecological traits would help to contextualize these homoplasious transitions and assess their functional significance. These results offer a more complete understanding of trait evolution in the family.

Aside from *Maranta* s.l., only *Saranthe* was recovered as monophyletic, with *S. klotzschiana* sister to remainder. *Saranthe klotzschiana* is distinguished by persistent inflorescence components longer than in other species (vs. caducous) [14]. Its position in the plastid tree (Figure S1) must be investigated in future studies. The other species were divided into two subclades, one including species with compound synflorescences, *S. madagascariensis* (Benth.) K.Schum and *S. composita* (Link) K.Schum., and the other those with simple synflorescences, *S. leptostachya* (Regel & Körn.) Eichle and *S. eichleri* Petersen [14]. Both *Ctenanthe* and *Stromanthe* were found to be non-monophyletic. *Stromanthe bahiensis* was sister to all other species in clade A, including *Saranthe* plus *Stromanthe* and *Ctenanthe*. Since its description by Yoshida-Arns et al. [11], this species has been considered aberrant in the genus, and, at the time of description, was compared to the also distinct *S. hjalmarssonii* (Körn.) Petersen ex K.Schum., an unsampled species from Central America. However, in morphological characteristics, *S. hjalmarssonii* is more like other species of *Stromanthe* than *S. bahiensis*. *Stromanthe bahiensis* bears longer petioles (≥14 cm vs. ≤8 cm), oblong to oblanceolate leaf blades (vs. elliptic), longer inflorescence peduncles with different vestiture (4–35 cm, glabrescent to hirsute vs. 1.8–7.6 cm, sparsely villous), and green spathes (vs. yellow). *Stromanthe hjalmarssonii* is morphologically like *S. jaquinii* (Roem. & Schult.) H.Kenn. & Nicolson, the latter included in our analyses.

Based on its strongly supported phylogenetic position and unusual morphology, we conclude a taxonomic change is necessary, for which two options are possible: (i) lump all species of *Ctenanthe* and *Saranthe* under *Stromanthe* or (ii) erect a new genus for *S. bahiensis*. The first option would require many new combinations, whereas the second involves a single change. We prefer the latter option. Although sharing with *Stromanthe* the caulescent ramified habit, lax florescences, strongly flexuous rachises, dolichoblastic cymules and no bracteoles [15], *S. bahiensis* is easily distinguished by longer cymules and unequal outer staminodes, both differing from the characters found in the remaining species of *Stromanthe* (Table 2). Furthermore, the similarities of *S. bahiensis* with the other *Stromanthe* species were reconstructed as plesiomorphic or homoplastic (Figure 3). Plesiomorphic and homoplastic traits have often led to artificial classifications, particularly in taxa established before the advent of molecular systematics [16]. Such misinterpreted traits can obscure evolutionary relationships, necessitating generic recircumscription.

Recognition of a new genus, *Dolichopoda*, is supported by our phylogenetic evidence, morphological distinctiveness, and the need for a classification reflecting evolutionary relationships. The ancestral state reconstructions provide an evolutionary explanation for the separation of *S. bahiensis* from *Stromanthe* and description of *Dolichopoda*. Our findings suggest that morphological evolution in the *Maranta* clade may have been shaped by homoplasy, which may partially explain the high morphological overlap observed among genera. This realignment not only resolves phylogenetic inconsistencies but also facilitates more accurate biogeographic and ecological inferences.

Although *Stromanthe* is not supported (PP 0.73), it includes *Ctenanthe dasycarpa*, a result previously found in other studies [3,8]. Here, it was recovered with strong support (PP 99) with *S. jacquinii* (Roem. & Schult.) H.Kenn. & Nicolson and *S. stromanthoides* (J.F.Macbr.) L.Andersson, which share many morphological characteristics with *C. dasycarpa, e.g., colored* spathes, diffuse axillary inflorescences, no bracteoles, and general distribution (northern South America, Panamá and Costa Rica) [5]. *Ctenanthe dasycarpa* also bears slightly flexuous rachises, a noteworthy characteristic of *Stromanthe*, but in *C. dasycarpa* these are not as pronounced as among the other species of the genus, which probably explains why it was placed in *Ctenanthe* [12]. We provide below a combination in *Stromanthe*, making both *Ctenanthe* and *Stromanthe* monophyletic and morphologically diagnosable (Table 1, see identification key below). The other species of *Stromanthe* form two clades, one is not supported (PP 0.60) with *S. tonckat* (Aubl.) Eichler, *S. porteana* Gris, S. glabra Yosh.-Arns and *S. schottiana* (Körn.) Eichler, which have concolorous leaves and simple to slightly ramified synflorescences. The other clade is strongly supported (PP 1.00) and includes *S. thalia* and *S. sanguinea*, species with highly ramified synflorescences and discolorous leaf blades [15]. *Stromanthe thalia* and *S. sanguinea* have been considered synonyms [17]. Although some relationships in *Stromanthe* were unsupported, the overall topology agrees with previous studies [3,10] and is morphologically consistent. Nevertheless, our proposed combination for *C. dasycarpa* is strongly supported. Further genomic evidence can provide more robust phylogenetic resolution for internal relationships of the genus.

In *Ctenanthe* (minus *C. dasycarpa*), *C. muelleri* is sister to all other sampled species of the genus. This species differs from the others of the genus by its monosymmetric florescences with secondary spathes (vs. asymmetric with distichous spathes in the other taxa) [15]. The other species are all characterized by congested florescences, straight rachises and brachyblastic cymules. One subclade, *C. glabra* (Körn.) Eichler, *C. luschnathiana* (Regel & Körn.) Eichler and *C. compressa* (A.Dietr.) Eichler, shares terminal-apical synflorescences and is sister to *C. casupoides* Petersen plus *C. oppenheimiana* (É. Morren) K. Schum with basal synflorescences. The other subclade, *C. marantifolia* (Vell.) J.M.A.Braga & H.Gomes, *C. amabilis* (É. Morren) H.Kenn. & Nicolson, *C. setosa* (Roscoe) Eichler, *C. burle-marxii* H.Kenn. and *C. kummeriana* (É.Morren) Eichler, shares discolorous leaves that generally are adaxially greyish and abaxially green or purple [15]. In our matrix, we could not include only three species of *Ctenanthe*, the recently described *C. brevibractea* F.Fraga & J.M.A.Braga from Espírito Santo [18], *C. ericae* L.Andersson, and *C. amphiandina* L.Andersson, the last two native to the Amazon Forest [5,15,18]. These species are morphologically like *C. muelleri* Petersen and *C. lanceolata* Petersen, which are rosulate herbs with lax terminal synflorescences of 2 or 3 nodes [15,18,19]. We summarize the main features distinguishing the five genera of the Maranta clade in Table 2 and provide a key to identify them.

### Taxonomic Treatment

***Dolichopoda*** N.Luna, L.P.Felix, E.Pessoa, ***gen. nov.***

**Figure 4:**
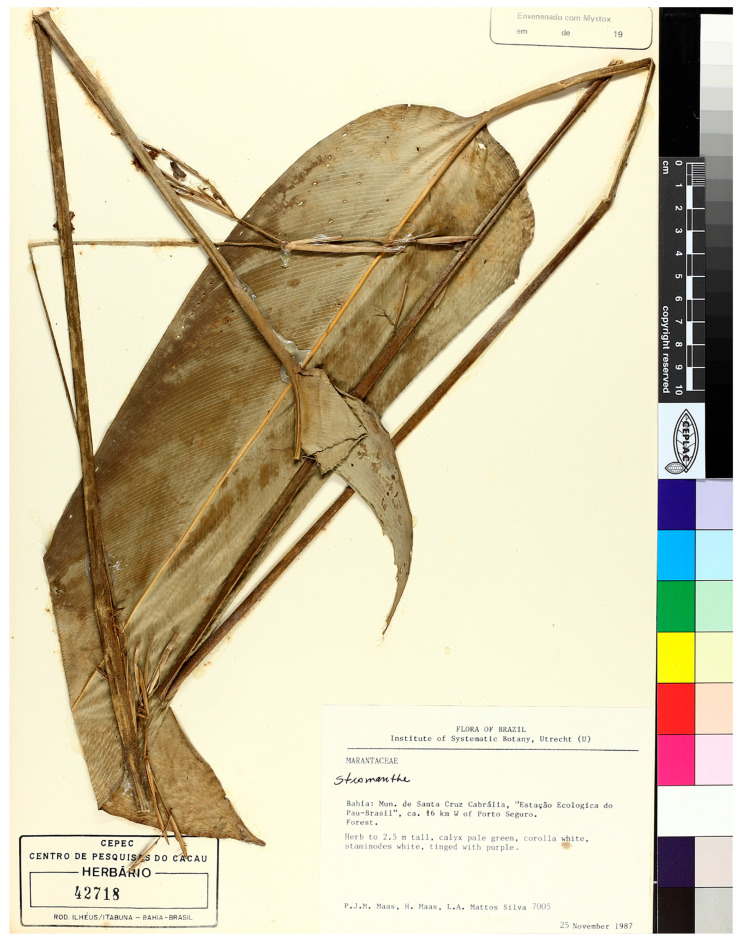
Type species: *Dolichopoda bahiensis* (Yosh.-Arns, Mayo & J.M.A.Braga) N.Luna (CEPEC42718) © Herbário do Centro de Pesquisas do Cacau. Reproduced with permission.

Type species: *Dolichopoda bahiensis* (Yosh.-Arns, Mayo & J.M.A.Braga) N.Luna, ***comb. nov.*** (≡*Stromanthe bahiensis* Yosh.-Arns, Mayo & J.M.A.Braga, Nordic J. Bot. 29: 357, 2011). (Figure 4).

Type: BRAZIL. Santa Cruz de Cabrália, Estação Ecológica do Pau Brasil, ca 16 km west of Porto Seguro, 25 November 1987, P.J.M. Mass et al. 7005 (holotype CEPEC 42718, isotypes: GB, RB, U).

Diagnosis: *Dolichopoda* is characterized by its caulescent erect habit with branched stems that bear long axillary synflorescences that are pendulous when mature, inflorescence units with lax spathes and strongly flexuous rachis, and dolichoblactic cymules. It is similar to *Stromanthe* but differs by its longer cymule peduncles (2.0–3.0 vs. 0.4–1.8 cm) and two unequal outer staminodes (vs. absent and when two, equal).

Description: Caulescent, branched herbs, 1.5–2.5 m long tall; rhizome slender; cataphylls to 40.0 cm long, lanceolate to ovate, green, glabrous, acute apically with ciliate margins; first internode of the aerial stem 0.5–1.6 m long, green, glabrous to hirsute. Leaves antitropic; leaf sheaths 25.0–67.0 cm long, green, glabrous to sparsely pillose, pubescent at margins; petiole 14.0–70.0 cm long, green, sparsely hirsute, slightly canaliculate; pulvinus 2.5–6.5 cm long, flat, green, glabrescent; leaf blade 30.0–68.0 × 8.0–30.0 cm, oblong to oblanceolate, concolorous green, glabrous, central vein glabrous to sparsely hirsute, rounded basally, acute and asymmetrical apically. Synflorescence axillary, 2–3-noded, subtended by a leafy bract; synflorescence prophyll 2.0–4.0 cm long, greenish, translucid, lanceolate to oblong, bicarinate, glabrous, acute apically; peduncle 1.5–76.0 cm long, green, glabrous to glabrescent, pendulous when mature. Inflorescence prophyll 1.0–5.0 cm long, greenish, translucid, lanceolate to oblong, bicarinate, glabrous, sometimes with hirsute ridges, acute apically; peduncle 4.0–35.0 cm long, green, glabrescent to hirsute, pendulous when mature; 1–4 florescences per node; florescence prophyll 0.5–6.7 cm long, greenish, translucid, lanceolate to oblong, bicarinate, glabrous, sometimes with hirsute ridges, acute apically; peduncle 2.0–18.0 cm, green, glabrous to hispidulous; rachis 7–35 cm long, strongly flexuous, lax, green, fully hispidous to hispiduous only at nodes; internodes between spathes 1.0–4.0 cm long, spathes 3–10, 1.5–5.0 × 0.1–0.2 cm, distichous, green, narrowly lanceolate to ovate, glabrous, deciduous, attenuate to acute or acuminate apically; cymules dolichoblastic, 2–4 per spathe; cymule peduncle 2.0–3.0 cm long, light green, glabrous; cymule prophyll 0.8–1.5 cm long, greenish, translucid, lanceolate to oblong, bicarinate, glabrous, acute apically. Flowers 2 per cymule; pedicel 0.1–0.8 cm long, light green, glabrous; sepals 0.5–0.6 × 0.1–0.2 cm, green, narrowly elliptic, glabrous, acute apically, deciduous; corolla white; corolla tube 0.2–0.4 cm long, straight, white, glabrous; corolla lobes 0.3–0.6 × 0.15–0.20 cm, elliptic to oblong, white, glabrous, round to obtuse apically; outer staminodes 2, unequal, the major 0.5–0.6 × 0.3–0.4 cm, the minor 0.5–0.6 × 0.2–0.25 cm, obovate, lilac, glabrous, round to emarginate apically; callose staminode 0.5–0.6 × 0.3–0.35 cm, oblong, glabrous, purplish white, round to emarginate apically, 1 conspicuous marginal, longitudinal callus; staminode cucullate 0.4–0.6 × 0.2–0.3 cm, oblong, glabrous, purplish white, round apically, revolute, distal appendix 0.1–0.15 cm long, deflexed; anther 0.1 cm long; petaloid appendix 0.3–0.4 cm long, oblong, white, emarginate apically; style 0.4–0.7 cm long; ovary 0.1–0.3 cm long, smooth, brown, pilose. Fruits 0.7 cm long, oblong, angular, wrinkled when dry, vinaceous, sparsely pilose.

Etymology: The name refers to the synflorescence morphology: *dolichos* Ancient Greek for long, and *poda*, Ancient Greek for foot, in this case, the peduncles.

**Figure 5:**
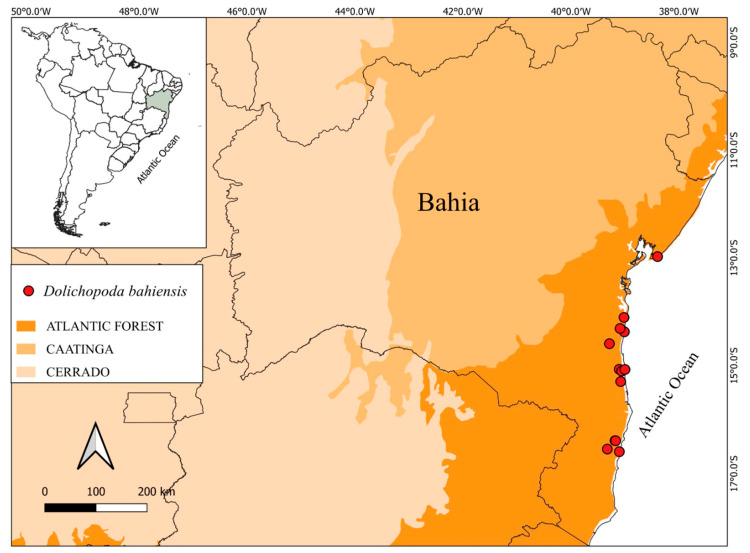
Distribution of *Dolichopoda bahiensis* (Yosh.-Arns, Mayo & J.M.A.Braga) N.Luna.

Geographic distribution and conservation: *Dolichopoda* is endemic to the Atlantic Forest of Brazil and known only from the southern portion of the State of Bahia (Figure 5). Only 12 populations of *D. bahiensis* are currently known. It is a light-tolerant species and grows in forest borders and open areas inside forest fragments and restingas. The threat status of this species was classified by Yoshida-Arns et al. [11] as vulnerable (VU) because land cover and land use of the native habitat has drastically changed, mainly by new housing and extensive livestock grazing. The flowering period appears to be throughout the year with observations in February, June, September and November; fruits observed in March.

Specimens examined: BRAZIL. Bahia: Santa Cruz de Cabrália, Reserva Biológica do pau- brasil, CEPLAC, 15 September 1971, Santos 1925 (CEPEC 7696); Ilhéus, ramal da faz. Ipiranga a 21 km de Olivença, 25 October 1972, Pinheiro 1934 (CEPEC 8930, RB 544860); Santa Cruz Cabrália, Arredores da estação Ecológica do Pau-brasil, ca. 17 km a W de Porto Seguro, 18 October 1978, Mori 10773 (CEPEC 15448); Santa Cruz de Cabrália, Reserva Biológica do Pau-Brasil, CEPLAC, 01 november 1983, Eupunino 17 (CEPEC 8164); Ilhéus, estrada Olivença/Maruim, entre os Km 7–10, 19 May 1985, Martinelli 111111 (CEPEC 37501, RB 233375); Santa Cruz de Cabrália, área da Estação Ecológica do Pau-Brasil (ESPAB), perto do KM 16 da Rod. Eunápolis/Porto Seguro (BR 367), ao longo da trilha para o córrego do Roncador, 18 February 1986, Andersson 1696 (CEPEC 38953); Porto Seguro, próximo a porteira, 4 July 1990, Folli, D.A. 1179 (CEN 57474), CVRD 2792); Ilhéus, entroncamento estrada Ilhéus/Uma, Vila Brasil, km 3, 19 September 1992, Coradin 8672 (CEN 21814, RB 437305); Porto Seguro, Reserva do Brasil Holanda de Ind. S/A, entrada no Km 22 da rod. Eunapólis/P.Seguro ca. 9.5 km na entrada, 6 April 1994, Carvalho 4502 (HUEFS 191423, RB 544889, SPF 232331); Maraú, ca. 8 km do entroncamento da estrada para Tremembé, 4 September 1999, Carvalho 6726 (RB 604569); Ilhéus, Acuípe, estrada para vila Brasil, 6 May 2000, Silva 386 (HUEFS 45356); Una, litoral sul, assentamento Vitorópolis, 25 June 2001, Santana 748 (ALCB 58066, CEPEC 109653); Porto Seguro, Estação Pau-Brasil, CEPLAC, trilha EPB 2, 18 January 2002, Almeida 54 (CEN 52001); Uruçuca, antiga estrada que liga Ubaitaba a Maraú, Fazenda água boa, 11 June 2006, Amorim 6055 (CEPEC 112301); Ca. 8 km do entroncamento da estrada de Tremembé, 5 August 2006, Carvalho 6726 (CEPEC 83230); Porto Seguro, Parque Nacional do Pau Brasil, 4 June 2009, Matos 1775 (CEPEC 126107, RB 604565); Porto Seguro, RPPN Estação Veracel, borda de Floresta alta, platô, 7 March 2010, Carvalho et al. 284 (ALCB 122440, CEPEC 127991); Porto Seguro, Trancoso, mata de tabuleiro, 12 September 2010, Folli 6727 (RB 854511, SAMES 3180); Porto Seguro, Reserva da Brasil Holanda S/A. Entrada no Km 22 Rod. Eunápolis/Porto Seguro ca. 9.5 km na entrada, 8 April 2011, Carvalho 4502 (CEPEC 61164); Salvador, região metropolitana Salvador, Av. Paralela, Green Ville, parcela 8, ind. 631, 10 May 2011, Guedes et al. 18214 (ALCB 100650); Porto Seguro, 12 September 2010, Folli 6727 (CVRD 13176); Itacaré, restinga da praia de itacarezinho, 17 October 2014, Lírio 1114 (RB 645763); Porto Seguro, RPPN Estação Veracel, setor 17, 24 March 2017, Santos s.n. (GCPP 00218); Porto Seguro, RPPN Estação Veracel, setor 5, 4 June 2017, Santos s.n., (GCPP 00316); Porto Seguro, Parque Nacional Pau Brasil, trilha córrego do jabuti, início da trilha, 6 November 2019, Fraga et al. 265 (RB 804254); Itacaré, RPPN Pedra do Sabiá, borda de mata, 14°18′29.6″ S 39°05′18.2″ W, 3 March 2023, Luna et al. 529 (EAN, HUEFS, RB, SAMES, UFP).

**New Combination**

*Stromanthe dasycarpa* (Donn.Smith) N.Luna, ***comb. nov.*** ≡ *Calathea dasycarpa* Donn.Smith). ≡ *Myrosma dasycarpa* (Donn.Smith) Woodson ≡ *Ctenanthe dasycarpa* (Donn.Smith) K.Schum.

Type: COSTA RICA. Comarca de Limón: Rio Hondo, Baia de Madre de Dios, “In Sykvis ad oras Rio Hondo”, elev. 200 m., November 1896 (fl.), Pitter 10350 (lectotype designated by Luna et al. [20]: CR 10350, digital image!).

Key for the Neotropical genera of the *Maranta* clade
1. Leaves homotropic..............................................................................................................................21’. Leaves antitropic................................................................................................................................32. Rachis slightly flexuous with obvious scars.........................................................................*Saranthe*2’. Rachis absent to straight without obvious scars.................................................................*Maranta*3. Florescence rachis strongly flexuous................................................................................................43’. Florescence rachis straight................................................................................................................54. Cymule peduncle ≥ 2 cm, unequal outer staminodes...................................................*Dolichopoda*4’. Cymule peduncle ≤ 1.8 cm, outer staminodes equal if two..........................................*Stromanthe*5. Bracteole present, corolla tube as long as wide, shorter than or equaling the sepals...*Ctenanthe*5’. Bracteole absent, corolla tube longer than wide, longer than sepals...............................*Maranta*

## 4. Materials and Methods

### 4.1. Sampling

This study included 40 species of the *Maranta* clade *sensu* Prince and Kress [8]. *Halopegia azurea* K.Schum. was selected as outgroup based on Prince and Kress [8], Al-Gharaibeh [10] and Fernandes et al. [3]. Our sampling included 14 species of *Maranta* s.l., 13 species in *Ctenanthe* (76%), five species in *Saranthe* (55%), and nine species in *Stromanthe* (ca. 50%). Our limited sampling for *Stromanthe* does not undermine our conclusions because most missing species are not Brazilian and based on morphology are unlikely to be related to *S. bahiensis*.

Specimens collected in the field (21 in total) were preserved in silica gel [21] for DNA extraction. Vouchers were deposited in UFP and EAN (Tables S1 and S2). Morphological data used for discussion were observed in herbarium and living specimens. The morphological descriptions were based on living material cultivated in the Experimental Garden of the Plant Cytogenetics Laboratory of the Center of Agricultural Sciences of the Federal University of Paraíba in addition to the herbarium material, and complemented with information from the pertinent literature [1,11,15,22,23,24,25,26,27].

### 4.2. DNA Extraction Amplification and Sequencing

Total DNA was extracted from 50 mg of silica-gel-dried tissue using the 2 × CTAB method [28]. Four molecular markers were selected based on their performance in previous phylogenetic studies of Marantaceae: the plastid markers *matK*, *trnL-F*, and *rps16* and the nuclear ribosomal ITS (nrITS: ITS1 spacer +5.8S gene +ITS2 spacer). A partial copy of *matK* was amplified using the primers 1639R, trnK2R, and 782F [29,30,31]. The primers C and F from Taberlet et al. [32] were used for the *trnL-F* spacer/intron region. The *rps16* intron was amplified with the primers of Oxelman et al. [33]. For ITS we used the primers of Schloötterer [34]. Amplification used a final volume of 25 μL: 12.5 μL of Dream TAq mix (Thermofisher, Waltham, MA, USA), 0.5 μL forward primer, 0.5 μL reverse primer, 10.5 μL H2O and 1 μL total DNA.

A specific program was used for each primer, with the following: *matK*, 94 °C for 1 min.; 30 cycles of 94 °C for 30 segs., 56 °C for 30 segs., 72 °C for 1 min; followed by a final extension at 72 °C for 7 min; *trnL-F*, 94 °C for 1 min.; 35 cycles of 94 °C for 30 segs., 53 °C for 40 segs., 72 °C for 40 segs.; followed by a final extension at 72 °C for 5 min; *rps16*, 95 °C for 3 min.; 35 cycles of 95 °C for 30 segs., 55 °C for 30 segs.; 72 °C for 45 segs., followed by a final extension at 72 °C for 5 min; nrITS, 94 °C for 3 min.; 35 cycles of 94 °C for 45 segs., 56 °C for 1 min., 72 °C for 2 min., followed by a final extension at 72 °C for 5 min.

The PCR products were then purified using AMpure XP type (Beckman Coulter, Brea, CA, USA) kit and sequenced with a BigDye^®^ Terminator v. 3.1 Cycle Sequencing kit (Applied Biosystems, Waltham, MA, USA), following the manufacturer’s protocols. Sequencing was performed with an ABI3730xl Genetic Analyzer (Applied Biosystems).

These molecular markers have produced good resolution and support in previous studies of Marantaceae [3,7,8,9,10]. Recently published studies have successfully established new genera in Zingiberales using comparable molecular datasets [2,35]. Despite the limitations of ITS in regard to hybridization scenarios and incomplete lineage sorting [36], it remains widely used for generic delimitation in angiosperms due to its high variability [3,37,38,39].

### 4.3. Alignment and Phylogenetic Analysis

Forward and reverse chromatograms were combined using Geneious Prime v.2021.1.1 (Biomatters, Auckland, New Zealand). The sequences generated in this study are available in GenBank and their accession numbers are listed in Table S2. Alignments used the MUSCLE [40] plugin implemented on Geneious. Bayesian inference (BI) used MrBayes v.3.2.7a [41] (implemented at the CIPRES Science Gateway portal, [42]) on separate nrITS and combined plastid regions, followed by a combined dataset. We checked gene tree incongruence using the incongruence length difference test [43] in PAUP* v.4.0a159 [44] with 1000 replicates. Substitution models for each region were selected using JModelTest v.2.1.10 [45] with the Bayesian information criterion (Table 1). We employed GTR+G for all regions, in two independent runs with four chains each with Markov chain Monte Carlo parameters (MCMC) defined for 40,000,000 generations sampling every 4000 trees. The first 2500 trees were discarded as burn-in (25%). Convergence between the runs were verified using Tracer v.1.6 looking for ESS values above 200 (Rambaut et al. 2018), and trees were edited using the software FigTree v.1.3 [46]. Clades with ≥0.95 posterior probabilities (PP) were considered supported [47,48,49].

### 4.4. Ancestral State Reconstruction

To assess whether key morphological traits used in taxonomic descriptions and generic delimitation can serve as diagnostic characters (synapomorphies) in the *Maranta* clade, we reconstructed ancestral character evolution using an ultrametric tree generated in BEAST 1.8.0 [50] of the combined plastid and nuclear dataset. The analysis employed a lognormal relaxed molecular clock model and a Yule process prior, running for 50 million generations with sampling every 5000 generations. We applied the best-fitting nucleotide substitution models (as described above) and retained default settings for other parameters. Convergence was verified in Tracer v1.6 [51], ensuring all ESS values exceeded 200, and the maximum clade credibility tree was summarized using TreeAnnotator v1.10.4 [50].

We compiled morphological data of four key features often used as diagnostic traits for species of the *Maranta* clade included in the analyses: (i) organization of the aerial shoot system (adapted from Costa et al. [52]), (ii) rachis flexuosity [15], (iii) spathe compactness (following Andersson [22]) and (iv) cymule type [22] (Table S3). These traits were the basis of *S. bahiensis* being described in *Stromanthe*. We then performed maximum likelihood ancestral character estimation for each discrete character separately, using the function ‘ace’ from the R package ‘ape’ [53], as implemented in RASP v.4.2 [54]. We evaluated the fit of three discrete trait models using the corrected Akaike Information Criterion: ER (equal rates model), where all transition rates were equal; SYM (symmetric model), where forward and reverse transitions were equal; and ARD (all rates different model), where all transition rates were distinct. The ER model was a better fit for all traits and the ancestral state reconstruction analyses were completed using default parameters.

## Figures and Tables

**Table 1:** Characteristics of each molecular marker, individually and combined, as well as nucleotide substitution models used for Bayesian inference.

	nrITS	*matK*	*rps16*	*trnL-F*	Combined Plastid	All Markers Combined
Taxon number	29	23	45	40	41	42
Alignment length	681	1602	902	948	3452	4133
Number of variable positions	226 (33.2%)	123 (7.5%)	136 (15%)	103 (10.8%)	345 (10%)	561 (13.6%)
Number of informative sites	109 (16%)	36 (2.2%)	49 (5.4%)	14 (1.5%)	95 (2.7%)	196 (4.7%)
Substitution model	TIM3+G	GTR+G	TPM2uf+G	TIM1	-	-

**Table 2:** Morphological characteristics to distinguish genera of the *Maranta* clade.

Genera/ Characters	*Dolichopoda*	*Ctenanthe*	*Maranta* s.l.	*Saranthe*	*Stromanthe*
Habit	Caulescent	Rosulate or caulescent	Rosulate, caulescent, zingiberoid or scandent	Rosulate	Caulescent
Synflorescence	Axillary, branched, lax, 1–4 florescence per node	Axillary or terminal-basal, simple to branched, congested, 1–4 florescences	Axillary, terminal-apical, or arising from rhizome, simple to branched, congested or lax 1–6 florescence per node	Axillary or terminal-basal, branched, congested, 1–3 florescence per node	Axillary or terminal-apical, simple to branched, lax, 1–4 florescences per node
Inflorescence penducle	4–35 cm long., pendulous	Sessile to 22 cm long, erect	Sessile to 18 cm long, erect	Sessile to 15 cm long, erect	0.7–22.5 cm long, erect
Spathe	Deciduous, chartaceous	Persistent, chartaceous or membranous	Persistent, chartaceous or membranous	Persistent or deciduous, membranous	Persistent or deciduous, membranous or papyraceous
Rachis	Strongly flexuous, hispid to hispid at nodes, 7–35 cm long	Straight, glabrous or pilose, 2–15 cm long	Absent, if present, straight, glabrous to glabrescent, puberules, hirsute to hirsute only at the base, 3–8 cm long	Slightly flexuous, puberulent to sericeous, 3–10 cm long.	Slightly to strongly f lexuous, glabrous, villous, lanuginous, 0.9–18.0 cm long,
Cymules	2–4 per spathe, dolichoblastic	2–7 per spathe, brachiblastic	1–6 per spathe, brachiblastic or dolichoblastic	1 per spathe, dolichoblastic	1–7 per spathe, brachiblastic to dolichoblastic
Bracteole	Absent	Present	Absent	Absent	Absent, rarely present
Outer staminodes	2 unequal	2 slightly unequal	2 equal or unequal	2 equal or unequal	Absent, 1–2 equal
Callose staminode	1 callus	1 to 2 calluses	1 or 3 calluses	1 or 2	1 callus
Sepals in fruits	Deciduous	Deciduous or persistent	Deciduous	Deciduous	Deciduous or persistent

## Data Availability

The sequences produced are available in Genbank (accession numbers available in Table S2 in the Supporting Information) the public repository of NCBI. Other supporting data are included as Supplementary Files.

## Supplementary Materials

The following supporting information can be downloaded at: https://www.mdpi.com/article/10.3390/plants14223486/s1. Table S1. GenBank accessions used in the analyses. Specimen vouchers are available at: “1” [7]; “2” [8]; “3” [29]; “4” [55]; “5” [10]; “6” [56]; “7” [57]; “8” [58]; “9” [59]; “10” [3]; “11” [60]; “12” sequenced here. Table S2. Voucher information of collected specimens used in the phylogenetic analyses. Table S3. Morphological data of five key features often used as diagnostic traits for the Maranta clade. Table S4. Ancestral states for the nodes indicated in Figure 3 following the codes available on Table S3. Figure S1. Phylogenetic relationships of Ctenanthe and related genera produced with Bayesian inference of one nuclear (rITS) (50% majority rule consensus tree) based on A. nuclear ITS, and B. plastid regions combined (rps16 intron, trnL-F intron and spacer and matK exon). Posterior probabilities are shown above the branches.

## Abbreviations

The following abbreviations are used in this manuscript:

PP	Posterior Probabilities
