# Supplementary material for: Molecular Phylogenetics and Morphological Analyses Support Dolichopoda, a New Neotropical Genus of Marantaceae (Zingiberales)
Source: Plants (Basel). 2025 Nov 15;14(22):3486. doi: 10.3390/plants14223486 (PMC12656207; doi:10.3390/plants14223486)
Supplement: Supplementary file 1 [file plants-14-03486-s001.zip › Table S1.pdf]

Table S1. GenBank accessions used in the analyses. Specimen vouchers are available at: "1" Andersson and Chase (2001); "2" Prince and Kress (2006); "3" Borchsenius et al. (2012); "4" [55]; "5" [10]; "6" [56]; "7" [57]; "8" [58]; "9" [59]; "10" [3]; "11" [60]; "12" sequenced here.

Species/marker	ITS	*mat*K	*rps*16	*trn*L-F
Ctenanthe amabilis (E. Morren) H. Kenn. & Nilcolson	KU215058[4]	–	KU214930[4]	KU215178[4]
Ctenanthe burle-marxii H. Kenn.	OL536433[9]	OL689946[8]	AF141041[1]	AY140367[2]
Ctenanthe casupoides Petersen	PV500580[12]	–	PV543543[12]	PV535778[12]
Ctenanthe compressa (A. Dietr.) Eichler	PV500581[12]	PV504835[12]	PV543544[12]	PV535779[12]
Ctenanthe glabra (Körn.) Eichler	–	PV504836[12]	PV543545[12]	PV535780[12]
Ctenanthe kummeriana Eichler	KU215056[4]	–	–	–
Ctenanthe luschnathiana (Regel & Körn.) Eichler	PV500582[12]	–	PV543546[12]	–
Ctenanthe lanceolata Petersen	PV500583[12]	–	–	PV535781[12]
Ctenanthe marantifolia (Vell.) J.M.A. Braga & H. Gomes	PV500584[12]	PV504837[12]	PV543547[12]	PV535782[12]
Ctenanthe muelleri Petersen	PV500585[12]	PV504838[12]	PV543548[12]	PV535783[12]
Ctenanthe oppenheimiana (E. Morren) K. Schum.	KU215059[4]	–	AF141043[1]	KU215179[4]
Ctenanthe setosa (Roscoe) Eichler	PV500586[12]	PV504839[12]	PV543549[12]	PV535784[12]
Dolichopoda bahiensis (Yosh.-Arns, Mayo & J.M.A. Braga) N. Luna	PV500587[12]	PV504840[12]	PV543550[12]	PV535785[12]
Halopegia azurea (K. Schum.) K. Schum.	KY214936[6]	AY140291[2]	AF141048[1]	KY214479[6]
Maranta arundinacea L.	JQ341260[3]	JQ341325[3]	ON227494[10]	JN413111[3]
Maranta cannifolia (L.) N. Luna & G. Fern.	ON221847[10]	–	AF141070[1]	ON642076[10]
Maranta cristata Nees & Mart.	AY673056[2]	AY140303[2]	AY656145[2]	KU215181[4]
Maranta furcata Nees & Mart.	–	–	PV543553[12]	PV535788[12]
Maranta gigantea N. Luna & E.M. Pessoa	–	–	ON239734[9]	ON677531[10]

Maranta hexantha (Poepp. & Endl.) D. Dietr.	–	AY140294[2]	PV543551[12]	AY140375[2]
Maranta hoffmanni (K. Schum.) G. Fernandes & N. Luna)	–	JQ588304[5]	AF141051[1]	–
Maranta leuconeura E. Morren	MF796520[7]	AY140303[2]	KU214935[4]	KU215182[4]
Maranta pohliana Körn.	ON228325[10]	–	ON239737[10]	ON642073[10]
Maranta polystachya (K. Schum.) J.M.A. Braga	–	PV504841[12]	PV543552[12]	PV535787[12]
Maranta protracta Miq.	–	–	AF141059[1]	ON703239[10]
Maranta orbiculata (Körn.) K. Schum.	–	–	AF141055[1]	AY140383[2]
Maranta ruiziana Körn.	ON221402[10]	–	AF141060[1]	ON703240[10]
Maranta sophiana Yosh.-Arns, F. Fraga & J.M.A. Braga	ON230019[10]	–	ON239738[10]	ON642074[10]
Saranthe composita (K. Koch) K. Schum.	PV500588[12]	PV504842[12]	PV543555[12]	PV535789[12]
Saranthe eichleri Petersen	PV500589[12]	–	PV543556[12]	PV535790[12]
Saranthe klotzschiana (Körn.) Eichler	PV500590[12]	PV504843[12]	–	–
Saranthe leptostachya (Regel & Körn.) Eichler	–	–	AF141080[10]	–
Saranthe madagascariensis (Benth.) K. Schum.	–	PV504844[12]	PV543557[12]	PV535791[12]
Stromanthe dasycarpa (Donn. Sm.) N. Luna & L.P. Felix	–	–	AF141042[1]	–
Stromanthe glabra Yosh.-Arns	PV500591[12]	PV504845[12]	PV543558[12]	PV535792[12]
Stromanthe jacquini (Roem. & Schult.) H. Kenn. & Nicolson	–	–	AF141087[1]	–
Stromanthe porteana Gris	PV500592[12]	PV504846[12]	PV543559[12]	PV535793[12]
Stromanthe sanguinea (Reider) Sond.	–	OP805594[11]	AF141089[1]	OP805594[11]
Stromanthe schottiana (Körn.) Eichler	–	PV504847[12]	PV543560[12]	PV535794[12]
Stromanthe stromanthoides (J.F. Macbr.) L. Andersson	–	MH603445[8]	MH603445[8]	MH603445[8]
Stromanthe thalia (Vell.) J.M.A. Braga	–	PV504849[12]	PV543562[12]	PV535795[12]
Stromanthe tonckat (Aubl.) Eichler	PV500593[12]	PV504848[12]	PV543561[12]	PV535796[12]
