# Supplementary material for: Molecular Phylogenetics and Morphological Analyses Support Dolichopoda, a New Neotropical Genus of Marantaceae (Zingiberales)
Source: Plants (Basel). 2025 Nov 15;14(22):3486. doi: 10.3390/plants14223486 (PMC12656207; doi:10.3390/plants14223486)
Supplement: Supplementary file 1 [file plants-14-03486-s001.zip › Table S2.pdf]

Table S2. Voucher information of collected specimens used in the phylogenetic analyses.

Species	Locality in Brazil	Collector name and number (herbarium)
Ctenanthe casupoides Petersen	Ibateguara, AL	*N. Luna 506* (UFP)
Ctenanthe compressa (A. Dietr.) Eichler	Caruaru, PE	*N. Luna 536* (UFP)
Ctenanthe glabra (Körn.) Eichler	Belém, PA	*L. Pessoa 20023* (EAN)
Ctenanthe lanceolata Petersen	Una, BA	*N. Luna 514* (UFP)
Ctenanthe luschnathiana (Regel & Körn.) Eichler	Chã Preta, AL	*N. Luna 510* (UFP)
Ctenanthe marantifolia (Vell.) J.M.A. Braga & H. Gomes	Conceição da Barra, ES	*N. Luna 554* (UFP)
Ctenanthe muelleri Petersen	Três Cachoeiras, RS	*N. Luna 574* (UFP)
Ctenanthe setosa (Roscoe) Eichler	Itacaré, BA	*N. Luna 530* (UFP)
Dolichopoda bahiensis (Yosh.-Arns, Mayo & J.M.A. Braga) N. Luna	Itacaré, BA	*N. Luna 529* (UFP)
Maranta furcata Nees & Mart.	Pinheiros, ES	*N. Luna 614* (UFP)
Maranta hexantha (Poepp. & Endl.) D. Dietr.	São Lourenço da Mata, PE	*N. Luna 601* (UFP)
Maranta polystachya (K. Schum.) J.M.A. Braga	Pindorama do Tocantins, TO	*L. P. Felix 19684* (EAN)
Saranthe composita (Link) K. Schum.	Conceição da Barra, ES	*N. Luna 553* (UFP)
Saranthe eichleri Petersen	Tuneiras do Oeste, PR	*H. Geraldino 188* (UFMT)
Saranthe klotzschiana (Körn.) Eichler	Itacaré, BA	*N. Luna 528* (UFP)
Saranthe madagascariensis (Benth.) K. Schum.	Caruaru, PE	*N. Luna 533* (UFP)
Stromanthe glabra Yosh.-Arns	Brejo da Madre de Deus, PE	*N. Luna 496* (UFP)
Stromanthe porteana Gris.	Recife, PE	*N. Luna 578* (UFP)
Stromanthe thalia (Vell.) J.M.A. Braga	Itacaré, BA	*N. Luna 525* (UFP)
Stromanthe tonckat (Aubl.) Eichler	Ibateguara, AL	*N. Luna 505* (UFP)
Stromanthe schottiana (Körn.) Eichler	Una, BA	*N. Luna 513* (UFP)
