# Supplementary material for: Molecular Phylogenetics and Morphological Analyses Support Dolichopoda, a New Neotropical Genus of Marantaceae (Zingiberales)
Source: Plants (Basel). 2025 Nov 15;14(22):3486. doi: 10.3390/plants14223486 (PMC12656207; doi:10.3390/plants14223486)
Supplement: Supplementary file 1 [file plants-14-03486-s001.zip › Table S3.pdf]

Table S3. Morphological data of five key features often used as diagnostic traits for the *Maranta* clade. Aerial shoot system adapted from Costa *et al.* (2011): A. Rosulate: unbranched, showing the spiral phyllotaxy of the leaves and reduced aerial stem; B. Caulescent: branched, with alternate distichous phyllotaxy and developed erect aerial stem; C. Caulescent decumbent: branched, with alternate distichous phyllotaxy and developed decumbent aerial stem; D. Zingiberoid: unbranched with alternate distichous phyllotaxy and reduced aerial stem.

Species	Aerial shoot system	Rachis flexuosity	Spathe compactness	Cymule type
Ctenanthe amabilis	Rosulate (A)	Straight (A)	Congested (A)	Brachyblastic (A)
Ctenanthe burle-marxii	Rosulate (A)	Straight (A)	Congested (A)	Brachyblastic (A)
Ctenanthe casupoides	Rosulate (A)	Straight (A)	Congested (A)	Brachyblastic (A)
Ctenanthe compressa	Rosulate (A)	Straight (A)	Congested (A)	Brachyblastic (A)
Ctenanthe glabra	Rosulate (A)	Straight (A)	Congested (A)	Brachyblastic (A)
Ctenanthe kummeriana	Rosulate (A)	Straight (A)	Congested (A)	Brachyblastic (A)
Ctenanthe lanceolata	Rosulate (A)	Straight (A)	Congested (A)	Brachyblastic (A)
Ctenanthe luschnathiana	Rosulate (A)	Straight (A)	Congested (A)	Brachyblastic (A)
Ctenanthe muelleri	Rosulate (A)	Straight (A)	Congested (A)	Brachyblastic (A)
Ctenanthe marantifolia	Caulescent (B)	Straight (A)	Congested (A)	Brachyblastic (A)
Ctenanthe oppenheimiana	Rosulate (A)	Straight (A)	Congested (A)	Brachyblastic (A)
Ctenanthe setosa	Rosulate (A)	Straight (A)	Congested (A)	Brachyblastic (A)
Dolichopoda bahiensis	Caulescent (B)	Strongly flexuous (B)	Lax (B)	Dolichoblastic (B)
Maranta arundinacea	Rosulate (A)	Straight (A)	Lax (B)	Dolichoblastic (B)
Maranta cannifolia	Rosulate (A)	Straight (A)	Congested (A)	Sub-brachyblastic (C)
Maranta cristata	Caulescent	Straight (A)	Lax (B)	Dolichoblastic (B)

Species	Aerial shoot system	Rachis flexuosity	Spathe compactness	Cymule type
	decumbent (C)			
Maranta furcata	Zingiberoid (D)	Straight (A)	Lax (B)	Dolichoblastic (B)
Maranta gigantea	Zingiberoid (D)	Straight (A)	Lax (B)	Dolichoblastic (B)
Maranta hexantha	Rosulate (A)	Straight (A)	Lax (B)	Dolichoblastic (B)
Maranta hoffmani	Rosulate (A)	Straight (A)	Lax (B)	Dolichoblastic (B)
Maranta leuconeura	Caulescent decumbent (C)	Straight (A)	Lax (B)	Dolichoblastic (B)
Maranta orbiculata	Rosulate (A)	Straight (A)	Congested (A)	Sub-brachyblastic (C)
Maranta pohliana	Caulescent (B)	Straight (A)	Lax (B)	Dolichoblastic (B)
Maranta polystachya	Rosulate (A)	Straight (A)	Lax (B)	Dolichoblastic (B)
Maranta protracta	Caulescent (B)	Straight (A)	Lax (B)	Dolichoblastic (B)
Maranta ruiziana	Caulescent (B)	Straight (A)	Lax (B)	Dolichoblastic (B)
Maranta sophiana	Caulescent (B)	Straight (A)	Lax (B)	Dolichoblastic (B)
Saranthe composita	Rosulate (A)	Slightly flexuous (C)	Congested (A)	Sub-brachyblastic (C)
Saranthe eichleri	Rosulate (A)	Slightly flexuous (C)	Congested (A)	Sub-brachyblastic (C)
Saranthe klotzchiana	Rosulate (A)	Slightly flexuous (C)	Congested (A)	Sub-brachyblastic (C)
Saranthe leptostachya	Rosulate (A)	Slightly flexuous (C)	Congested (A)	Sub-brachyblastic (C)
Saranthe madagascariensis	Rosulate (A)	Slightly flexuous (C)	Congested (A)	Dolichoblastic (B)
Stromanthe dasycarpa	Caulescent (B)	Slightly flexuous (C)	Congested (A)	Brachyblastic (A)
Stromanthe glabra	Caulescent (B)	Strongly flexuous (B)	Lax (B)	Sub-brachyblastic (C)
Stromanthe jacquini	Caulescent (B)	Slightly	Lax (B)	Sub-brachyblastic (C)

Species	Aerial shoot system	Rachis flexuosity	Spathe compactness	Cymule type
		flexuous (C)		
Stromanthe porteana	Caulescent (B)	Strongly flexuous (B)	Lax (B)	Sub-brachyblastic (C)
Stromanthe sanguinea	Caulescent (B)	Strongly flexuous (B)	Lax (B)	Sub-brachyblastic (C)
Stromanthe schottiana	Caulescent (B)	Strongly flexuous (B)	Lax (B)	Sub-brachyblastic (C)
Stromanthe stromathoides	Rosulate (A)	Slightly flexuous (C)	Lax (B)	Sub-brachyblastic (C)
Stromanthe thalia	Caulescent (B)	Strongly flexuous (B)	Lax (B)	Sub-brachyblastic (C)
Stromanthe tonckat	Caulescent (B)	Strongly flexuous (B)	Lax (B)	Dolichoblastic (B)
