# Supplementary material for: Molecular Phylogenetics and Morphological Analyses Support Dolichopoda, a New Neotropical Genus of Marantaceae (Zingiberales)
Source: Plants (Basel). 2025 Nov 15;14(22):3486. doi: 10.3390/plants14223486 (PMC12656207; doi:10.3390/plants14223486)
Supplement: Supplementary file 1 [file plants-14-03486-s001.zip › Table S4.pdf]

**Table S4.** Ancestral states for the nodes indicated in Figure 3 following the codes available on Table S3.

| | Aerial shoot system | Rachis flexuosity | Spathe compactness | Cymule type |
|---|---|---|---|---|
| 1 | A = 99.62% | C = 99.93% | A = 99.69% | B = 49.03% |
| 2 | A = 99.18% | C = 99.86% | A = 99.37% | C = 99.34% |
| 3 | A = 99.15% | C = 99.86% | A = 99.32% | C = 94.37% |
| 4 | A = 96.50% | C = 99.45% | A = 97.52% | C = 96.31% |
| 5 | B = 99.65% | B = 99.94% | B = 99.72% | C = 99.71% |
| 6 | B = 99.93% | B = 99.98% | B= 99.94% | C = 99.94% |
| 7 | B = 99.96% | B = 99.99% | B = 99.96% | C = 99.96% |
| 8 | B = 99.82% | B = 99.97% | B = 99.86% | C = 68.95% |
| 9 | B = 99.61% | B = 99.93% | B = 99.68% | C = 97.82% |
| 10 | A = 49.66% | C = 99.99% | A = 50% | A = 49.81% |
| 11 | B = 97.62% | C = 99.98% | B = 96.59% | C = 97.44% |
| 12 | B = 98.53% | B = 66.23% | B = 98.74% | C = 98.57% |
| 13 | A = 48.41% | A = 99.94% | A = 99.74% | A = 99.73% |
| 14 | A = 94.69% | A = 99.95% | A = 99.79% | A = 99.79% |
| 15 | A = 99.13% | A = 99.92% | A = 99.63% | A = 99.61% |
| 16 | A = 99.43% | A = 99.91% | A = 99.57% | A = 99.56% |
| 17 | A = 99.94% | A = 99.98% | A = 99.95% | A = 99.95% |
| 18 | A = 99.90% | A = 99.98% | A = 99.91% | A = 99.91% |
| 19 | A = 99.70% | A = 99.94% | A = 99.76% | A = 99.75% |
| 20 | A = 99.82% | A = 99.97% | A = 99.85% | A = 99.85% |
| 21 | A = 99.20% | A = 99.86% | A = 99.38% | A = 99.37% |

| | Aerial shoot system | Rachis flexuosity | Spathe compactness | Cymule type |
|---|---|---|---|---|
| 22 | A = 99.82% | A = 99.97% | A = 99.84% | A = 99.85% |
| 23 | A = 97.59% | A = 99.61% | A = 98.29% | A = 98.11% |
| 24 | A = 64.22% | A = 74.80% | A = 65.72% | A = 65.27% |
| 25 | A = 95.15% | C = 76.64% | A = 93.87% | C = 84.93% |
| 26 | A = 77.26% | C = 54.29% | A = 77.37% | C = 73.40% |
| 27 | A = 97.66% | A = 99.61% | B = 98.30% | B = 98.15% |
| 28 | A = 97.99% | A = 99.70% | B = 70.57% | B = 69.65% |
| 29 | A = 97.69% | A = 99.62% | A = 50% | B = 47.70% |
| 30 | A = 71.22% | A = 99.77% | B = 84.82% | B = 86.48% |
| 31 | C = 62.06% | A = 99.52% | B = 93.95% | B = 94.86% |
| 32 | A = 80.14% | A = 99.71% | B = 92.92% | B = 94.31% |
| 33 | B = 96.60% | A = 99.44% | B = 97.60% | B = 97.34% |
| 34 | A = 48.30% | A = 99.93% | B = 99.70% | B = 99.69% |
| 35 | B = 64.12% | A = 99.65% | B = 98.19% | B = 98.12% |
| 36 | B = 46.25% | A = 99.68% | B = 98.58% | B = 98.46% |
| 37 | B = 82.93% | A = 99.55% | B = 97.86% | B = 97.71% |
| 38 | B = 89.04% | A = 99.75% | B = 98.61% | B = 98.63% |
| 39 | B = 45.05% | A = 99.74% | B = 97.89% | B = 98.21% |
| 40 | A = 73.77% | A = 88.09% | B = 80.75% | B = 76.79% |
