# Supplementary figures and images for: Molecular Phylogenetics and Morphological Analyses Support Dolichopoda, a New Neotropical Genus of Marantaceae (Zingiberales)
Source: Plants (Basel). 2025 Nov 15;14(22):3486. doi: 10.3390/plants14223486 (PMC12656207; doi:10.3390/plants14223486)

A

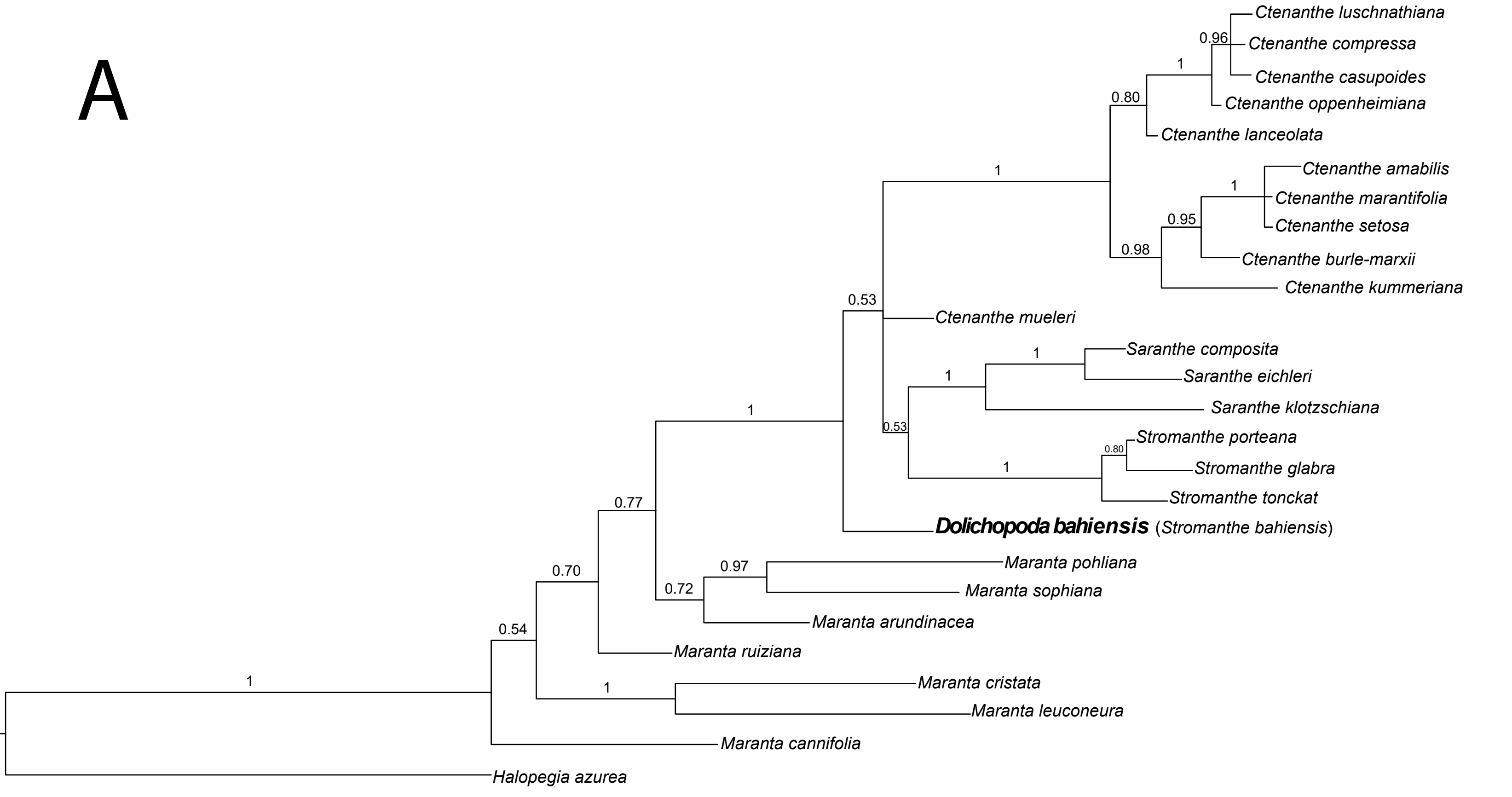

B

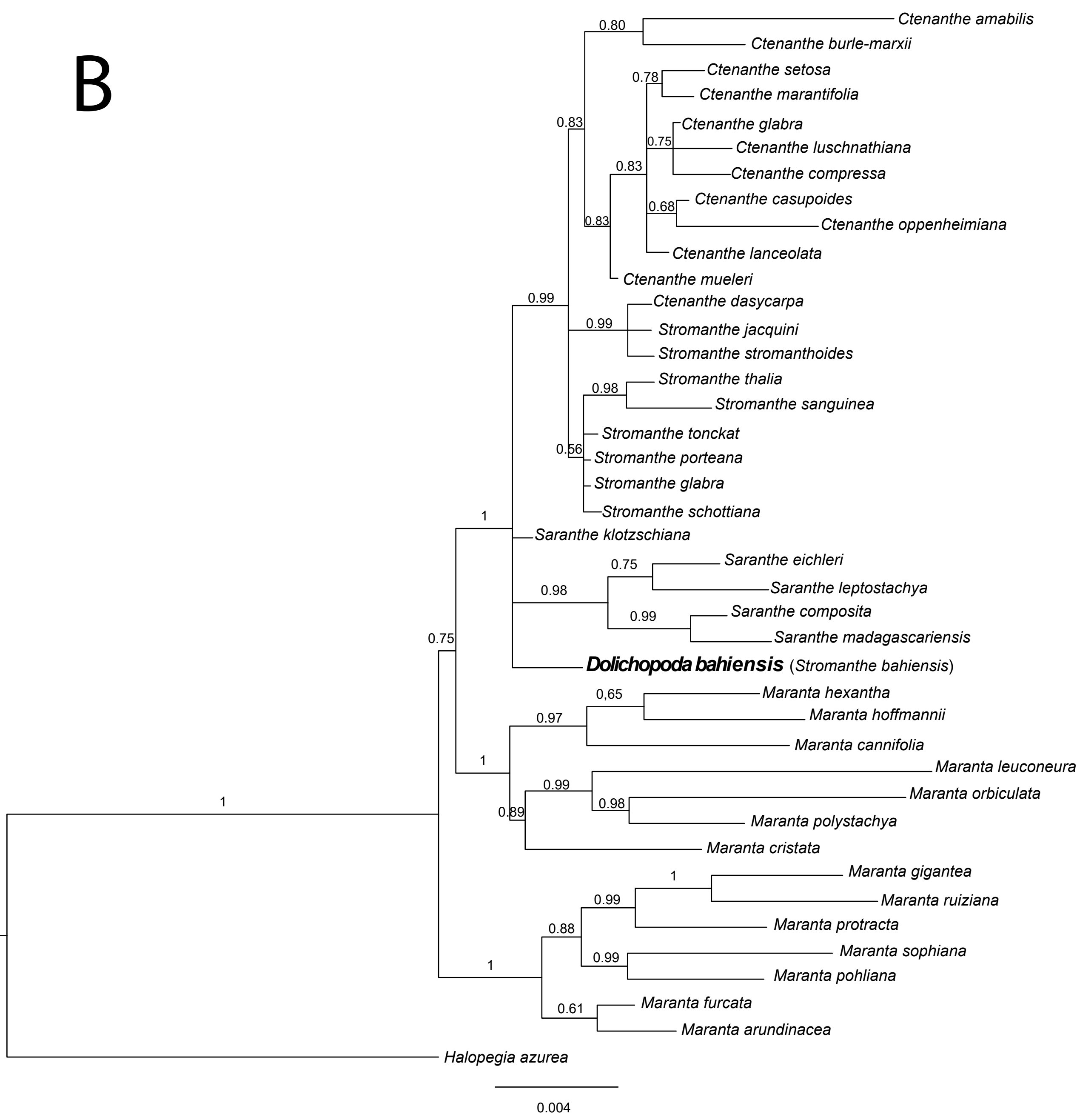

Supplement: Supplementary file 1 [file plants-14-03486-s001.zip › Figure S1.pdf]
